# Perspectives of Healthcare Professionals Regarding Factors Associated with Antimicrobial Resistance (AMR) and Their Consequences: A Cross Sectional Study in Eastern Province of Saudi Arabia

**DOI:** 10.3390/antibiotics10070878

**Published:** 2021-07-19

**Authors:** Mohamed A. Baraka, Amany Alboghdadly, Samar Alshawwa, Asim Ahmed Elnour, Hassan Alsultan, Taha Alsalman, Hussain Alaithan, Md. Ashraful Islam, Kareem Ahmed El-Fass, Yehia Mohamed, Abdulsalam A. Alasseri, Khairi Mustafa Fahelelbum

**Affiliations:** 1 Clinical Pharmacy Program, College of Pharmacy, Al Ain Campus, Al Ain University, Abu Dhabi P.O. Box 64141, United Arab Emirates; assahura2021@gmail.com; 2 Clinical Pharmacy Department, College of Pharmacy, Al-Azhar University, Cairo P.O. Box 11751, Egypt; 3 Department of Clinical Pharmacy, School of Pharmaceutical Sciences, Universiti Sains Malaysia, Penang P.O. Box 11800, Malaysia; amanyalboghdadly@gmail.com; 4 Pharmaceutical Sciences Department, College of Pharmacy, Princess Nourah bint Abdulrahman University, Riyadh P.O. Box 84428, Saudi Arabia; 5 Pharmacy Practice Department, College of Clinical Pharmacy, Imam Abdulrahman Bin Faisal University, Dammam P.O. Box 34212, Saudi Arabia; hassanalsultan3@gmail.com (H.A.); tahaalsalman@gmail.com (T.A.); hussain.aleithan@gmail.com (H.A.); maislam@iau.edu.sa (M.A.I.); 6 Department of Pharmacy Practice, College of Clinical Pharmacy, King Faisal University, Al-Hufuf P.O. Box 31982, Saudi Arabia; kelfass@kfu.edu.sa; 7 Department of Pathological Sciences, College of Medicine, Ajman University, Ajman P.O. Box 346, United Arab Emirates; y.mohamed@ajman.ac.ae; 8 Department of Microbiology and Immunology, Faculty of Pharmacy Boys, Al-Azhar University, Cairo P.O. Box 11751, Egypt; 9 Pharmacy Services Department, King Fahd Hospital of the University (KFHU), Imam Abdulrahman Bin Faisal University, Khobar P.O. Box 2208, Saudi Arabia; aaasseri@iau.edu.sa; 10 Pharmaceutical Sciences Program, College of Pharmacy, Al Ain University, Abu Dhabi P.O. Box 64141, United Arab Emirates; khairi.mustafa@aau.ac.ae

**Keywords:** antimicrobial resistance, AMR, Infections, antibiotics, inappropriate prescribing, healthcare professionals, education, training, antimicrobial stewardship programs, continuous professional development

## Abstract

Factors reported in the literature associated with inappropriate prescribing of antimicrobials include physicians with less experience, uncertain diagnosis, and patient caregiver influences on physicians’ decisions. Monitoring antimicrobial resistance is critical for identifying emerging resistance patterns, developing, and assessing the effectiveness of mitigation strategies. Improvement in prescribing antimicrobials would minimize the risk of resistance and, consequently, improve patients’ clinical and health outcomes. The purpose of the study is to delineate factors associated with antimicrobial resistance, describe the factors influencing prescriber’s choice during prescribing of antimicrobial, and examine factors related to consequences of inappropriate prescribing of antimicrobial. A cross-sectional study was conducted among healthcare providers (190) in six tertiary hospitals in the Eastern province of Saudi Arabia. The research panel has developed, validated, and piloted survey specific with closed-ended questions. A value of *p* < 0.05 was considered to be statistically significant. All data analysis was performed using the Statistical Package for Social Sciences (IBM SPSS version 23.0). 72.7% of the respondents have agreed that poor skills and knowledge are key factors that contribute to the inappropriate prescribing of antimicrobials. All of the respondents acknowledged effectiveness, previous experience with the antimicrobial, and reading scientific materials (such as books, articles, and the internet) as being key factors influencing physicians’ choice during antimicrobial prescribing. The current study has identified comprehensive education and training needs for healthcare providers about antimicrobial resistance. Using antimicrobials unnecessarily, insufficient duration of antimicrobial use, and using broad spectrum antimicrobials were reported to be common practices. Furthermore, poor skills and knowledge were a key factor that contributed to the inappropriate use and overuse of antimicrobials, and the use of antimicrobials without a physician’s prescription (i.e., self-medication) represent key factors which contribute to AMR from participants’ perspectives. Furthermore, internal policy and guidelines are needed to ensure that the antimicrobials are prescribed in accordance with standard protocols and clinical guidelines.

## 1. Introduction

Infectious diseases are caused by microorganism’s penetration into the epidermis, dermis, soft tissues of the skin, and other body tissues, leading to infectious diseases and comorbidities that can ultimately lead to sepsis and death [1]. Antimicrobial agents have the advantage of treating infectious diseases, and they have the potential not only to retain the quality of life of the patient but have also proved to be lifesaving in a variety of severe infectious conditions [2,3]. The improper use of antimicrobials has led to the development of antimicrobial resistant (AMR), which has been associated with a failure in managing infections, increased length of hospital stay, increased mortality, morbidity [4,5,6], and health costs [7,8]. Several factors have been described in the literature to be correlated with the inappropriate prescribing of antimicrobials [9] such as physicians with less experience, level of knowledge, uncertain diagnoses, and patient caregiver influences on physician’s decisions [10,11,12]. Over two million infections and 23,000 deaths annually in the United States were due to AMR [13]. Studies from diverse settings estimate that between 25% and 50% of antibiotic use in hospitals was suboptimal or unnecessary [14,15]. Gulf Cooperation Council countries face challenges of emerging AMR with no clear regional guidelines for antimicrobial use or specific policies for restricting and monitoring antimicrobial prescribing [16]. Monitoring AMR is critical for identifying emerging resistance and developing, monitoring, and evaluating the effectiveness of mitigation strategies [17,18]. Improvement in antimicrobial prescribing would minimize the risk of AMR, reduce healthcare costs, and improve patient clinical outcomes. In an attempt by the World Health Organization (WHO) to reduce AMR, several initiatives and programs have been established to educate healthcare providers on appropriate antimicrobial prescribing for evidence-based indications [19,20]. A study conducted in Saudi Arabia has revealed that the medications most commonly used were antibiotics [21]. In another study, it has been confirmed that certain types of medications, such as chemotherapeutic agents and antipsychotics, are exclusively prescribed by the specialists involved, while antibiotics are vigorously prescribed by all physicians as well as by allied health practitioners, irrespective of their knowledge or experience about antibiotics prescribed [22]. Accordingly, general practitioners are the most common prescribers of antibiotics in Saudi Arabia, relative to specialists and residents. The objectives of this study were to delineate the factors associated with antimicrobial resistant, describe the factors influencing prescriber’s choice during prescribing of antimicrobial, and examine the factors related to consequences of inappropriate prescribing of antimicrobial agents.

## 2. Results

A total of 184 healthcare providers have responded to the survey with a 96.8% response rate. Results have demonstrated that more than half of the respondents (53.8%) were pharmacists, (35.8%) were physicians, and (10.9%) were nurses (56% were males). Nearly 56% of the respondents’ age was less than 30 years, around one-third (30.4%) were in the age strata from 31 to 40 years, and only 13.6% were aged ≥41 years. Regarding years of practice, 37% of the respondents had less than 3 years of clinical experience, whereas only 16% reported that their experience exceeded 10 years. The majority (72.8%) have worked more than 8 h per day. Regarding the average of continuous professional development (CPD), (71%) of the respondents had more than 20 CPD hours per year (Table 1).

In the current study, six factors with various questions (including yes/no responses) on antimicrobial dispensing and prescribing practices were analyzed. At first, we assessed the factors influencing physicians’ choice during antimicrobial prescribing, which was reflected by eight questions. Results revealed that for the first question, “Parent/patient’s demand”, 32.6% have responded positively. For the second question, “Reading scientific materials (such as books, articles, and internet)”, 56.0% have responded positively as well. Responses retrieved from the third question, “Attending courses and lectures” were positively reported in 36.4%. Nearly half of the respondents (46.2%) agreed with the fourth question, “Cost of antibiotic”, while (59.8%) agreed with the fifth question, “Effectiveness and previous experience with the prescribed antimicrobial. Only 23.4% responded yes for the sixth question, “Recommendation by other colleagues”, while (39.7%) responded yes to the seventh question, “The knowledge gained during undergraduate or postgraduate training”. Nearly half (49.5%) of the respondents have agreed with the eighth question, “Availability of the antibiotic in the hospital formulary” (Table 2).

Findings revealed that a maximum of 72.7% of the respondents have agreed that poor skills and knowledge are a key factor which contributes to the inappropriate prescribing of antimicrobials. However, nearly half of the respondents (47.5%) have concurred that unrestricted availability of antimicrobials, inadequate supervision (45.4%), overworked/busy health care providers (32.2%), lack of physicians’ interest in the subject of antimicrobial prescribing (41.5%), and a lack of microbiology lab facilities (43.7%) were the major causes of inappropriate use of antimicrobial agents (Table 3).

Concerning the causes of AMR, the highest number of respondents (80.4%) concurred that using antimicrobial when they were not necessary, whereas not completing the full course of antimicrobials (59.8%) and using antimicrobials without physician prescription as self-medication (66.3%) were reported as the key factors which contribute to AMR (Table 4).

Regarding the factors detecting the important consequences of antimicrobial overuse, the results have shown that the majority (78.8%) of respondents agreed that it will lead to AMR. The inverse results found for the question “Reduce the duration of hospitalization” have shown that 79.9% of respondents disagreed with this statement. Furthermore, 85.9% of respondents didn’t believe that better patient outcomes can be a consequence of antimicrobial overuse. (54.9%) of respondents have denied that waste of resources and recurrence of infections (56.5%) can be the consequence of antimicrobials overuse (Table 5).

In order to assess the factors that may help to control AMR, nine questions were used. Results demonstrated that the question about “Physician education” reported a higher proportion (66.8%) of respondent’s agreement. On the other hand, for the question of “Consulting with infectious diseases experts” 55.4% of respondents agreed, and 58.2% have believed that providing local antimicrobial guidelines will contribute to the control of AMR. However, only 56% agreed with the statement that providing knowledge of pathogens and antimicrobial susceptibility test results will contribute to control AMR. The respondents have rejected the assumptions that obtaining local AMR profiles, practicing antimicrobial restriction, removing catheters when not essential, and targeting antimicrobial therapy to likely pathogens could contribute to controlling AMR (51.6%), (59.2%), (66.3%), and (70.1%), respectively (Table 6).

The survey’s final theme was designed to know about the most common resources used by healthcare providers for knowledge towards antimicrobials. Slightly above half (57.6%) of respondents have agreed that the Lexicomp/Sanford guide/up-to-date or other online resources were common resources. Most respondents, on the other hand, did not agree with other common resources, such as national/Saudi guidance, hospital/internal guidelines, smartphone medical applications, Red Book, Quick reference guides or booklets or medical journals, and other guidelines by professional organizations, infectious diseases specialists/service in the hospital, colleagues or seniors from your own team or specialty. The clinical pharmacists and medical representatives have not been considered as a source of information for antimicrobials Table 7.

## 3. Discussion

Our study documented the perceptions of healthcare professionals working in six tertiary hospitals in the eastern province of Saudi Arabian tertiary hospitals. The current study has delineated the factors associated with antimicrobial resistance (AMR), described the factors influencing prescriber’s choice during prescribing antimicrobials, and examined the factors related to the consequences of inappropriate prescribing of antimicrobials.

This study revealed that around half of the respondents acknowledged the importance of previous experience with antimicrobials, and reading scientific materials as contributing factors influencing physicians’ choice during antimicrobial prescribing. Conforming with a study conducted in Jeddah city in Saudi Arabia, our results confirmed their study findings [2]. Interestingly, attending courses and lectures was not considered a factor that may help control AMR. This might explain the poor knowledge among our respondents about the importance of attending courses and lectures to improve their antimicrobial practices [23,24].

Similar to healthcare professionals from other parts of the world, 72.7% of the respondents agreed that poor skills and knowledge were a key factor that contributes to the inappropriate use of antimicrobials [2,25]. However, most of the respondents have agreed that unrestricted availability of antimicrobials, overworked/busy health care providers, and lack of physicians’ interest in the subject of antimicrobial prescribing and infection management were not causes of the inappropriate prescribing of antimicrobials.

Many healthcare providers in our study have agreed that using antimicrobials when they were not necessary, not completing the full course of antimicrobials, and using broad spectrum antimicrobials were common practices. Further, using antimicrobials without physician prescription (self-medication) were identified as the key factors which contribute to AMR [23,26]. Our findings lend support to previously published studies [2,21,25]. In response to this, and based on the current study results, this can be controlled via increasing the sessions of CPD for healthcare providers on appropriate antimicrobial treatment, providing local antimicrobial guidelines and consulting with experts on infectious diseases. The majority of healthcare professionals have believed that AMR was a consequence of antimicrobial overuse [27,28,29]. On the other hand, they have denied that the waste of resources and recurrence of infections can be the consequences of antimicrobials overuse. We highly encourage each hospital to generate their internal policy or guidelines to guide and restrict antimicrobials′ overuse. In response to this, and based on healthcare professionals’ responses here, this can be rectified via CPD awareness programs and education on appropriate antimicrobial therapy, providing local antimicrobial guidelines, consulting with infectious diseases experts, and (above all) implementing antimicrobial stewardship programs. Yusef, D. et al. concluded that antimicrobial stewardship programs (ASPs) helped to reduce the use of antifungals in hospitalized patients and highlighted the importance of promoting antifungal stewardship among antibiotic stewardship [30]. Additionally, Rodolfo E. Quirós et al. showed significant improvement in antimicrobial utilization after multiple and intensive ASPs [5]. Interestingly, targeting antimicrobial therapy to likely pathogens was not mostly considered a factor that may help control AMR. This may be due to the poor of knowledge in our respondents on the importance of targeting pathogens. Some recent studies conducted in Saudi Arabia by Al-Harthi and colleague were in line with our results, wherein they identified the importance of healthcare professional education on appropriate antimicrobial therapy, providing local antimicrobial guidelines, and consulting with infectious diseases experts as crucial factors that can control AMR and its consequences. [8]

In fact, further studies are needed to target the reasons behind the gap in knowledge, experience, and skills on antimicrobials, as revealed by the current study respondents. This will help in controlling AMR and its consequences in our region.

### Limitations of the Study

There are some limitations to be considered when interpreting the study findings. First, although the research was conducted at six hospitals in the Eastern province that were well-resourced, the findings of the study may not be generalizable to other regions in Saudi Arabia. Second, participants were selected randomly and the characteristics of those who did not participate in the survey are unknown. Furthermore, purposive sampling was used to target doctors, nurses, and pharmacists from departments with high antibiotic use. Therefore, there are departments (such as psychiatry, for example) that are underrepresented. It is possible, therefore, that the sampling approach and recruitment limit the generalizability of the study results to other departments or hospitals. Finally, the sample included in the study was small, with a moderate response rate. We believe the results from the current study will be valuable in guiding educational efforts in order to prevent antimicrobial resistance and well received by clinicians in their respective institutions. However, our study’s strength lies in the fact that, to the best of our knowledge, it is the first study that investigates healthcare providers’ perceptions regarding understanding factors associated with AMR, their consequences, and possible ways to control such a problem.

## 4. Materials and Methods

The study was a cross-sectional study among health care providers (190) in six tertiary hospitals (4 governmental and 2 private hospitals with more than 500 beds) in the Eastern province of Saudi Arabia.

### 4.1. Sample Size

A published statistical formula was used to calculate the sample size for the unknown nature of population size. Finally, the sample was adjusted for potential missing or non-response error using the formula:

$$n=\frac{{\left(Z_{1-\beta }\right)}^{2}\left[p(1-p)\right]}{d^{2}}$$

where, *n* is the required sample size; *Z*_1 − *β*_ = *Z* is the value at power 1 − *β* (at power 90% this value is 1.24); *p* is the preferred population proportion (0.5); *d* is the margin of error (ideal value is 0.05); and $\;\;n_{1}=n/\left(1-e\right)$, where ‘*n*’ is the required sample size as per formula, ‘*n*_1_’ is the adjusted sample size, and ‘*d*’ is the potential missing or non-response rate. A minimum number of 190 samples was required to achieve 90% power when considering a 5% margin of error and a 10% missing/non-response error rate. Participants responses with multiple missing values have been removed from the SPSS database before analysis. The proportion of responses with missing data ranged from 5% to 7% across all questions in Part B.

### 4.2. Data Collection Instrument and Quality Assurance

#### Survey Development

Survey questions have been adopted and compiled from previously validated questionnaires from the published articles during literature review phase [6,8,10,11,12]. The initial version of the structured survey was developed after in-depth literature review to assess healthcare providers’ perceptions regarding understanding factors associated with AMR, their consequences, and possible ways to control such problem. The research panel (three pharmacists, statisticians, physicians, and the director of clinical pharmacy services) developed the survey and divided it into two parts with closed ended questions. This expert advisory panel finalized the survey and assured the quality after addressing the comments and corrections.

The survey was subdivided into two parts. The first (part A) including respondents’ characteristics and experiences. These characteristics included respondents’ gender, age, nationality, and city of residence in addition to their professional status, country of last professional degree, daily working hours, years of practice and average continuous medical education hours earned per year. The second part (part B) has described the factors related to antimicrobial prescribing and dispensing practices. This has contained a total of six factors with different options (wherein one is allowed to choose multiple options). These factors include factors influencing the choice during antimicrobial prescribing, factors that might be identified as causes of inappropriate antimicrobial use, causes of antimicrobial resistance, possible consequences of antimicrobial overuse, and factors that might help control antimicrobial resistance and the most common resources used for knowledge toward antimicrobials. In addition to evaluating the viability of the survey, the survey has been piloted to ensure face validity and has resolved both improvements in terminology and ease of use. However, the data collected from the pilot study were not involved in the final analysis. In terms of reliability, a variety of factors have been taken into account when planning this analysis to reduce the risk to the reliability. This has included that the data collection process was clearly documented and research procedures were followed as per the data collection protocol during the research process. Furthermore, the results were obtained on one occasion to simplify the capture of answers from participants and to help mitigate any unintended bias in the analysis of responses; close-ended questions were purposely selected for this survey design.

### 4.3. Administration of the Survey

A standard set of the pre-tested validated survey was used to collect data among health care providers (physicians, pharmacists, and nurses) by self-administrative procedure. In addition, questions for online surveys (QuestionPro) have been distributed to the hospital administration, pharmacy directors, and nursing supervisors via e-mails and related social media accounts to boost data collection. The data collection procedure was administrated by the investigators via several necessary visits, reminders by phone calls, and emails.

### 4.4. Statistical Analysis

The online survey (QuestionPro) has enabled the view of some missing cases among the data set. Therefore, we have used the ‘last-observation-carried-forward method’. Some of these cases were treated, and the rest were cleaned from the final analysis. Additionally, an informal technique was used to check and cleaned the whole data set. Variables were coded and recoded as per the requirements of study objectives. Descriptive statistics were utilized for data analysis. Frequencies and their corresponding percentages were reported to describe the factors and their respective items. A value of *p* < 0.05 was considered to be statistically significant. The whole data analysis was conducted using the Statistical Package for Social Sciences (IBM SPSS version 23.0).

## 5. Conclusions

The current study has identified comprehensive education and training needs for healthcare providers about the AMR and how to prevent such a problem. Using antimicrobials unnecessarily, insufficient duration of antimicrobial use, and using broad spectrum antimicrobials were all reported to be common practices. Furthermore, poor skills and knowledge were identified as a key factor that contributed to the inappropriate use and overuse of antimicrobials. Using antimicrobials without physician prescription (i.e., self-medication) were the key factors which contribute to AMR from participants’ perspectives. This problem can be controlled via increasing the sessions of CPD for healthcare providers on appropriate antimicrobial treatment, providing local antimicrobial guidelines and consulting with experts on infectious diseases. More studies about local AMR patterns are deemed necessary, and more efforts are warranted to educate clinicians about antimicrobial resistance. We highly encourage each hospital to generate their internal policy or guidelines to guide and restrict antimicrobials′ overuse to combat AMR.

## Figures and Tables

**Table 1:** Descriptive statistics for socio-demographic characteristics of respondents (*n* = 184).

Characteristics	Group	Frequency (*n*)	Percentage (%)
Gender	Male	103	56.0
	Female	81	44.0
Age (year)	Less than 30	103	56.0
	31–40	56	30.4
	≥41	25	13.6
Nationality	Saudi	154	83.7
	Non-Saudi	30	16.3
Region	Eastern of Saudi Arabia	178	96.7
	Others	6	3.3
Profession	Nurses	20	10.9
	Physicians	65	35.3
	Pharmacists	99	53.8
Country of last professional degree	Saudi Arabia	149	81.0
	Others	35	19.0
Working hours (per day)	Less than 8 h	50	27.2
	8 h and more	134	72.8
Years of Practice	Less than 3 years	68	37.0
	3–6 years	50	27.2
	6–10 years	36	19.6
	More than 10 years	30	16.3
Average of CME (continuous medical education) hours/year	Less than 20	53	28.8
	20 and above	131	71.2

**Table 2:** Factors influencing physicians’ choice during antimicrobial prescribing.

Items	Short Item Name	Responses	
		Yes (*n* & %)	No (*n* & %)
Parent/patient’s demand	PD	60 (32.6)	124 (67.4)
Reading scientific materials (such as, books, articles, and internet)	RSM	103 (56.0)	81 (44.0)
Attending courses and lectures.	ACL	67 (36.4)	117 (63.6)
Cost of antibiotic	CA	85 (46.2)	99 (53.8)
Effectiveness and previous experience with the drug antimicrobial	EEDA	110 (59.8)	74 (40.2)
Recommendation by other colleagues	RC	43 (23.4)	141 (76.6)
The knowledge gained during undergraduate or postgraduate training	KT	73 (39.7)	111 (60.3)
Availability of the antibiotic in the hospital formulary	AAHF	91 (49.5)	93 (50.5)

**Table 3:** Factors related to important causes of inappropriate prescribing of antimicrobial.

Items	Short Item Name	Responses	
		Yes (*n* & %)	No (*n* & %)
Poor skills and knowledge	PSK	133 (72.7)	50 (27.3)
Unrestricted availability of antimicrobials	UAA	87 (47.5)	96 (52.5)
Inadequate supervision	IS	83 (45.4)	100 (54.6)
Lack of physician interest in the subject of antimicrobial prescribing and infection management	LPI	76 (41.5)	107 (58.5)
Lack of effective hospital policies	LHP	85 (46.4)	98 (53.6)
Overworked/busy health care providers	OHP	59 (32.2)	124 (67.8)
Lack of microbiology lab facilities (lack of antimicrobial susceptibility test results)	LML	80 (43.7)	103 (56.3)

**Table 4:** Factor associated with the causes of antimicrobial resistant.

Items	Short Item Name	Responses	
		Yes (*n* & %)	No (*n* & %)
Using antimicrobial when they are not necessary	UANN	148 (80.4)	36 (19.6)
Not completing the full course of antimicrobial	NCFA	110 (59.8)	74 (40.2)
Using the same antimicrobial with a different brand	USADB	47 (25.5)	137 (74.5)
Using antimicrobials without physician prescription (self-medication)	UAWPP	122 (66.3)	62 (33.7)
Using broad spectrum antimicrobial, as a common practice	UBSA	94 (51.1)	90 (48.9)
Patient pressure for antibiotics as part of treatment	PPAT	77 (41.8)	107 (58.2)
Patients are able to buy antibiotics over-the-counter	PABA	80 (43.5)	104 (56.5)

**Table 5:** Factor related to important consequences of antimicrobial overuse.

Items	Factor Short Name	Responses	
		Yes (*n* & %)	No (*n* & %)
Antimicrobial resistant	AMR	145 (78.8)	39 (21.2)
Adverse drug reactions and medication errors	ADRME	78 (42.4)	106 (57.6)
Reduce duration of hospitalization	RDH	37 (20.1)	147 (79.9)
Better patient outcome	BPO	26 (14.1)	158 (85.9)
Waste of resources	WR	83 (45.1)	101 (54.9)
Recurrence of infections	RI	80 (43.5)	104 (56.5)

**Table 6:** Factors that may help to control antimicrobial resistant.

Items	Factor Short Name	Responses	
		Yes (*n* & %)	No (*n* & %)
Treating infection, not contamination or colonization	TINCC	74 (40.2)	110 (59.8)
Physician education on appropriate antimicrobial therapy	PEAAT	123 (66.8)	61 (33.2)
Consulting with infectious diseases experts	CIDE	102 (55.4)	82 (44.6)
Providing local antimicrobial guidelines	PLAG	107 (58.2)	77 (41.8)
Knowledge of pathogens and antimicrobial susceptibility test results	KPASR	103 (56.0)	81 (44.0)
Obtaining local antibiotic resistant profiles	OLARP	89 (48.4)	95 (51.6)
Practicing antimicrobial restriction	PAR	75 (40.8)	109 (59.2)
Removing catheters when not essential	RCNE	62 (33.7)	122 (66.3)
Targeting antimicrobial therapy to likely pathogens	TALP	55 (29.9)	129 (70.1)

**Table 7:** Factors associated with most common resources used for knowledge toward antimicrobials.

Items	Factor Short Name	Responses	
		Yes (*n* & %)	No (*n* & %)
National/Saudi guidelines	NG	83 (45.1)	101 (54.9)
Hospital/internal guidelines	HG	78 (42.4)	106 (57.6)
Lexicomp/Sanford guide/UPTODATE or other Online resources	LG	106 (57.6)	78 (42.4)
Smart phone medical applications	SMA	80 (43.5)	104 (56.5)
Red Book, Quick reference guides or booklets or Medical journals	RBRJ	41 (22.3)	143 (77.7)
Other guidelines by professional organizations (IDSA, CDC,…etc.)	OG	62 (33.7)	122 (66.3)
Infectious diseases specialists/service in the hospital	IDSH	66 (35.9)	118 (64.1)
Colleagues or seniors from your own team or specialty	CTS	36 (19.6)	148 (80.4)
Hospital/Clinical pharmacists or Drug information centres	HPGIC	62 (33.7)	122 (66.3)
Medical/Pharmaceutical representatives	MR	31 (16.8)	153 (83.2)
Others	Others	9 (4.9)	175 (95.1)

## Data Availability

Data will be available upon request.
